# Comparison of the Different Anesthesia Strategies for Atrial Fibrillation Catheter Ablation: A Systematic Review and Meta-Analysis

**DOI:** 10.1155/2022/1124372

**Published:** 2022-03-20

**Authors:** Naidong Pang, Jia Gao, Nan Zhang, Binghang Zhang, Rui Wang

**Affiliations:** ^1^The First Clinical Medical College, Shanxi Medical University, Taiyuan, Shanxi, China; ^2^Department of Cardiology, First Hospital of Shanxi Medical University, Taiyuan, Shanxi, China

## Abstract

**Background:**

Catheter ablation has become a widely applied intervention for treating symptomatic atrial fibrillation (AF), which can be performed under general anesthesia (GA), deep sedation, or conscious sedation (CS). But the strategy of anesthesia remains controversial.

**Objectives:**

This systematic review and meta-analysis aims to compare the advantages of GA/deep sedation and CS in AF catheter ablation, including procedural parameters and clinical outcomes.

**Methods:**

PubMed, Embase, and the Cochrane Library were searched up to November 2021 for randomized controlled trials and observational studies that assessed the outcomes of catheter ablation under GA/deep sedation or CS. Ten studies were included in this meta-analysis after screening with the inclusion and exclusion criteria. Heterogeneity between studies was evaluated by the I^2^ index and the Cochran *Q* test, respectively; sensitivity analysis including meta-regression was performed if heterogeneity was high. Publication bias was assessed using a funnel plot and Egger' test.

**Results:**

This meta-analysis found GA/deep sedation to be associated with a lower recurrence rate of AF catheter ablation (*p*=0.03). In terms of procedural parameters, there was no significant difference between the two groups for the procedural time (*p*=0.35) and the fluoroscopy time (*p*=0.60), while the ablation time was shorter in the GA/deep sedation group (*p*=0.008). The total complication rate and the incidence of serious adverse events were statistically insignificant between the two groups (*p*=0.07 and *p*=0.94). Meta-regression did not suggest any covariates as an influential factor for procedural parameters and clinical outcomes.

**Conclusion:**

GA/deep sedation may reduce the risk of recurrence after AF ablation without increasing the incidence of complications. GA/deep sedation shortens the ablation duration, although there is no statistical difference in other procedural parameters between GA/deep sedation and CS.

## 1. Introduction

Atrial fibrillation (AF) is one of the most common human arrhythmias and is associated with a number of serious diseases including stroke and heart failure. According to the American Heart Association estimates, the current prevalence of AF is between 2 and 4% in adults [1], with almost 6% in people over 65 [2]. Catheter ablation (CA) has been a guidelines-based treatment for AF [3], which is significantly more effective in terms of reducing arrhythmia recurrence rate than antiarrhythmic medication [4]. CA is a complex procedure that requires the patient to remain motionless for several hours and burns heart muscles; as a consequence, patients may experience anxiety and pain. However, in consideration of the safety operation of catheter, CA requires patients to remain “relatively quiet.” Therefore, anesthesia is essential for radiofrequency ablation of AF.

The CA of AF is usually performed under general anesthesia (GA), deep sedation, or conscious sedation (CS), depending on the preference and experience of operator and the general condition of the patient [5–7]. Patients with GA completely lost consciousness and are intubated and ventilated under positive pressure. The anesthesia depth of deep sedation approached GA. In addition, deep sedation may require assistance to maintain a patent airway but generally does not require endotracheal intubation. However, the patient could respond purposefully to verbal commands during CS and endotracheal intubation are not required to maintain a patent airway [8]. The GA and deep sedation have similar sedation depth and can keep the patient motionless; there is no clear line between the two strategies in this respect [9]. The main difference is airway management and anesthetic dosage [10], whereas if airway reflexes are not preserved during deep sedation, the same airway management as under GA should be performed. Regarding the use of anesthetic and sedative drugs, the sedative that is used the most frequently in the GA/deep sedation is propofol, while the most commonly used is midazolam in CS. The most commonly used opioid drug was remifentanil or fentanyl, regardless of the anesthesia strategy used [11]. In addition, GA also requires muscle relaxants.

Currently, some studies have shown that GA or deep sedation appears to improve the success rate of single ablation and reduce the prevalence of pulmonary vein reconnection [12, 13], while the opposite conclusion was drawn in other studies [14, 15]. They found GA not only has a higher recurrence rate but also brings more complications and operation time. In theory, in addition to patient comfort, GA/deep sedation contributes to better stability and lesion formation, which has also been determined in several studies [16, 17]. But longer anesthesia preparation time and anesthesia-related complications may be required [18]. Although several studies have compared the effects of different anesthesia strategies on AF catheter ablation, it remains unclear whether this is a conclusion due to the limited sample size and different research results. Therefore, we conducted a new systematic review and meta-analysis to verify the results of GA/deep sedation and CS techniques on AF ablation and illustrate which approach could provide more clinical benefits.

## 2. Methods

### 2.1. Data Sources and Search Strategy

Electronic databases including PubMed, Embase, and the Cochrane Library were searched for studies from the inception of each database through November 2021 by two independent reviewers (NP and JG). Search keywords included “atrial fibrillation,” “catheter ablation,” “radiofrequency ablation,” “pulmonary vein isolation,” “conscious sedation,” “deep sedation,” and “general anesthesia” without language restrictions. A combination of MeSH words and free words was used to search to improve accuracy and comprehensiveness. Furthermore, a manual search of additional literature was conducted by checking the reference lists of relevant research studies and review articles. We refer to the data retrieval and collection methods of similar studies [19]. Any discrepancies were arbitrated by a third reviewer (RW).

### 2.2. Inclusion and Exclusion Criteria

The following inclusion criteria were applied: (a) the design included randomized controlled trials (RCTs) and observational studies in clinical; (b) related to GA/deep sedation or CS in atrial fibrillation catheter ablation; and (c) contained periprocedural parameters and long-time clinical outcomes, including recurrence rates, operative time parameters, complication rates, and so on. Included studies should have stable follow-up. Published paper was excluded if it was a review, case report, or animal study. The included studies were not limited by patient's race, sex, age and countries where the studies were conducted.

### 2.3. Data Extraction and Quality Assessment

Original articles were obtained for each study, and relevant information was collected and entered into a prespecified spreadsheet. The data extracted from each eligible study were as follows: (a) study information (first author's name, year of publication, country where study was performed, sample size, ablation strategy, follow-up way and follow-up duration); (b) characteristics of participants (mean age, sex, atrial fibrillation type, and background disease); and (c) periprocedural parameters, efficacy indexes, and complications (procedure time, ablation time, fluoroscopy time, procedural success rate, all-cause death, pericardial effusion, and cardiac tamponade).

Regarding quality assessment, RCTs were assessed with the Cochrane Collaboration recommending tool, focusing on randomization method, allocation concealment, blind method, data integrity, reporting bias, and so on [20]. The quality assessment of observational studies was performed using the Newcastle-Ottawa Quality Assessment Scale (NOS). The point score system evaluated the categories of study participant selection, comparability of the results, and quality of the outcomes. The scores varied from zero to nine based on the quality of studies. Papers were graded as poor or common quality if they met <7 criteria and good if they met ≥7 criteria. Details of the NOS quality assessment are shown in Table 1. Two independent reviewers conducted data extraction and quality assessment (NP and JG). Any disagreements were adjudicated by a third reviewer (RW).

### 2.4. Statistical Analysis

The Review Manager (RevMan, version 5.3, the Nordic Cochrane Centre, the Cochrane Collaboration, Copenhagen) and Stata (version 12.0) were used for this meta-analysis. Statistical significance was set as a *p* value less than 0.05. Tests of heterogeneity were conducted by calculating *I*^*2*^, with a value greater than 50% considered to indicate significant heterogeneity. We also used the Cochran *Q* test to assess heterogeneity between studies and defined the indication of heterogeneity as *p* value less than 0.1 [21]. When heterogeneity was present, sensitivity analysis was performed to inspect the effect of a single study on the overall risk estimate by sequentially omitting one study at a time. Furthermore, meta-regression analysis was conducted to explore the sources of differences among studies. We assessed the publication bias by constructing a funnel plot and assessed funnel plot asymmetry by Egger's test.

## 3. Results

### 3.1. Search Results

**Figure 1:**
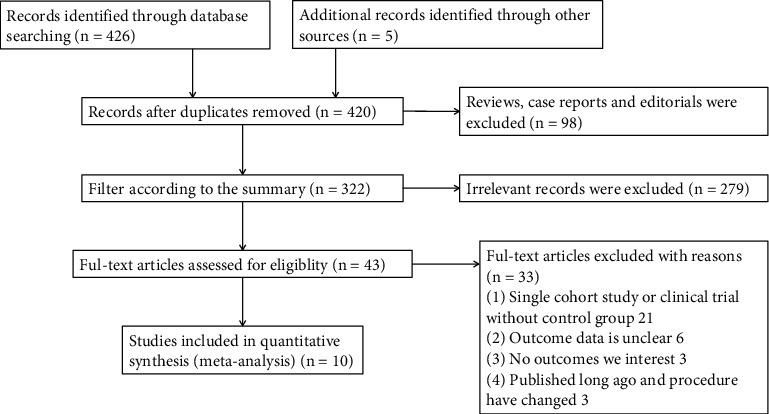
Flow diagram for study identification and inclusion.

Four hundred and twenty-six articles were initially identified from a literature search, and five additional studies were identified through the manual search of reference lists from these articles. Eleven duplicate studies were removed, and 98 reviews, case reports, and editorials were excluded. After screening the title and abstract of the remaining studies, 43 were retrieved for full-text review. Of these, 30 studies were further excluded because the index data were not available or there was no control group. In addition, three studies published too long ago were excluded because ablation techniques and materials have changed and lack guidance for current clinical practice. The remaining 10 studies, comprising two RCTs [22, 23] and 8 observational studies [13–15, 17, 24–27] satisfied the inclusion criteria and were eventually included in this meta-analysis. The literature search was conducted by two independent researchers (NP and JG).  Figure 1 shows the study flowchart.

### 3.2. Study Characteristics

The included studies comprised 2418 patients and were conducted in centers across China, the United Kingdom, Monaco, the Czech Republic, Bulgaria, and Japan. The mean age of the participants was 61.2, consisting of mainly male patients (70.5%). Regarding the ablation strategy, all studies used radiofrequency energy to perform pulmonary vein isolation, and additional ablation was performed as appropriate. Six studies included PAF and PsAF, three included PAF only, and one included PsAF only. Nine of the 10 studies reported anesthetic protocols specifically. The anesthetic protocols in the GA/deep sedation group mainly involved with intravenous anesthetics like propofol and opioid narcotics such as fentanyl, sufentanil, or remifentanil. Only one study used propofol and flunitrazepam for deep sedation. Furthermore, all studies with GA used muscle relaxants such as rocuronium and performed endotracheal intubation, whereas oxygen was delivered by nasal cannula in studies using deep sedation. The CS group was mainly treated with sedatives such as midazolam, small doses of fentanyl, and local anesthetics such as lidocaine or articaine. The follow-up duration ranged from 6 to 27 months and nine of the studies were longer than 12 months (90.0%). The mean follow-up duration was 12.4 months. Eight of the included studies screened the postoperative recurrence rate through 12-lead electrocardiogram (ECG) and 24-hour Holter surveillance at different follow-up times. Four studies also used 7-day ambulatory ECG monitoring or cardiac event recorders to screen for recurrence. In two studies, the follow-up method was not described. Detailed characteristics of all included studies are presented in Table 2. The analysis data in this meta-analysis include mean procedural time, mean fluoroscopy time, mean ablation time, postoperative recurrence rate, total complication rate, and serious complication rate. The procedural time was defined as the time from the patient entering the operating room to leaving at the end of the operation. The ablation time was defined as the time from the beginning of ablation to the completion of the whole ablation procedure.

### 3.3. Quality Assessment

**Figure 2:**
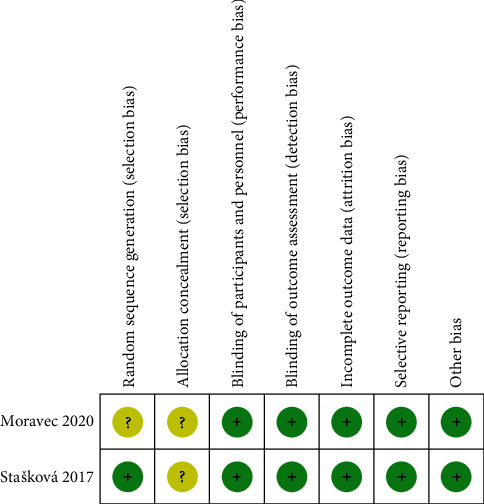
Risk of bias of included RCTs in the meta-analysis.

The two RCTs were of high quality according to the Cochrane Collaboration's criteria, although the allocation concealment in both studies was unclear.  Figure 2 shows the results from the risk of bias assessment of the RCTs. Observational studies were graded as good quality papers if the NOS scores were between 7 and 9 points, indicating that they were suitable for analysis (Table 2).

### 3.4. Evaluation of Perioperative Parameters of Different Anesthetic Strategies

#### 3.4.1. Mean Procedural Time

**Figure 3:**
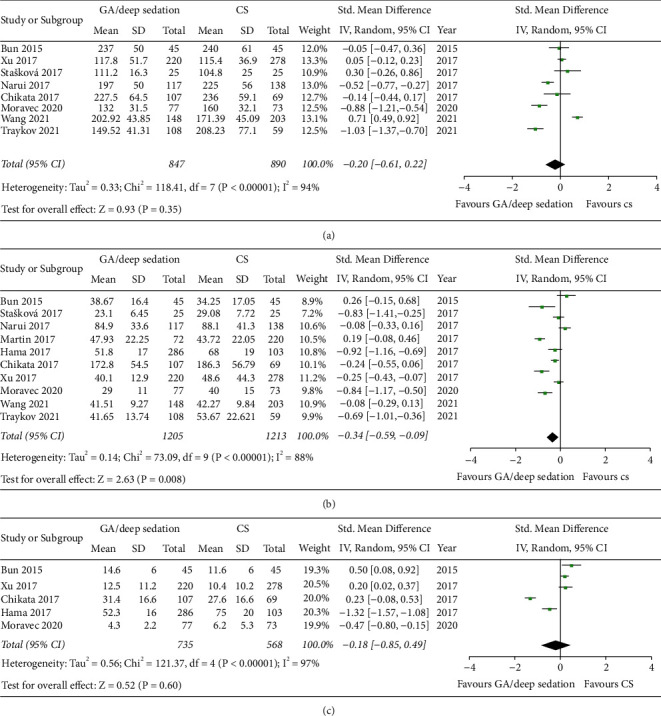
Forest plots comparing (a) mean procedural time of AF ablation, (b) mean ablation time, and (c) mean fluoroscopy time between the GA/deep sedation group and the CS group.

**Figure 4:**
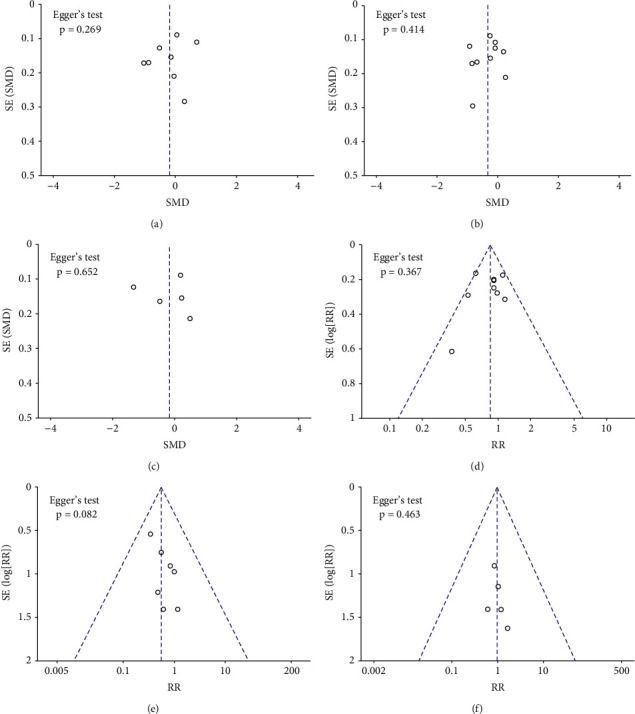
Funnel plots and Egger's test were used to assess publication bias of (a) mean procedural time, (b) mean ablation time, (c) mean fluoroscopy time, (d) postoperative recurrence rate, (e) total complication rate, and (f) incidence of serious adverse events.

Nine out of ten studies reported mean procedural time in catheter ablation of AF patients using either GA/deep sedation or CS. Of these, one study cannot be incorporated into the quantitative synthesis, which due to standard mean differences was not reported and could not be calculated. In the other eight studies, three studies favored the use of CS, while five studies found GA/deep sedation could shorten the procedural time. After quantitative synthesis, there is not significantly different between the two approaches on mean procedural time (SMD: −0.20, random-effect model, 95% CI, −0.61 to 0.22, *p*=0.35; Figure 3(a)). *I*^*2*^ was 94%, and the *p* value of the *Q* test was less than 0.00001, indicating a high degree of heterogeneity. A sensitivity analysis was performed that excluded one study at a time, but the overall heterogeneity was not significantly altered. No significant publication bias was observed in funnel plot and Egger's test (Figure 4(a)).

#### 3.4.2. Mean Ablation Time

Ten studies reported mean ablation time. Of these, one study reported the total radiofrequency energies of the two groups rather than the time, but it was still able to be used for comparison. Two studies favored the use of CS, while eight studies found that GA/deep sedation led to a shorter ablation time. Pooled analysis showed ablation time in GA/deep sedation is shorter and reached statistical significance (SMD: −0.34, 95% CI, −0.59 to −0.09, *p*=0.008; Figure 3(b)). A high degree of heterogeneity (*I*^*2*^ = 88%) was observed. Sensitivity analysis was conducted but could not reduce the overall heterogeneity, which by excluding one study at a time. No significant publication bias was observed in funnel plot and Egger's test (Figure 4(b)).

#### 3.4.3. Mean Fluoroscopy Time

A total of five studies provided fluoroscopy time data. Similarly, with procedural time, it was also found to be shorter with GA/deep sedation for mean fluoroscopy time in AF ablation, but did not reach statistical significance (SMD: −0.18, 95% CI, −0.85 to 0.49, *p*=0.60; Figure 3(c)). *I*^*2*^ was 97%, which means a high degree of heterogeneity. No significant publication bias was observed in funnel plot and Egger's test (Figure 4(c)).

### 3.5. Effect of Anesthesia Strategy on the Postoperative Recurrence Rate of AF

**Figure 5:**
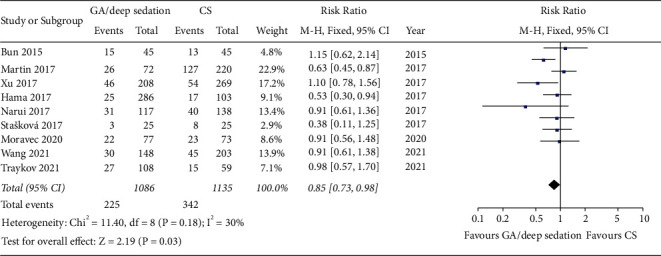
Forest plots comparing recurrence rates post-AF ablation between the GA/deep sedation group and the CS group.

Nine out of ten studies reported recurrence rates after a mean follow-up of 12.2 months after AF ablation. One study presented only the Kaplan–Meier curves but did not report the specific recurrence rate, so it could only be qualitatively analyzed. Pooled analysis of the included AF studies demonstrated that patients who used GA/deep sedation tended to have a lower risk of recurrence on follow-up compared with the CS group, and the result reached statistical significance (RR: 0.85, fixed-effect model, 95% CI, 0.73 to 0.98, *p*=0.03; Figure 5). A low degree of heterogeneity (*I*^*2*^ = 30%, *p*=0.18 of the *Q* test) was observed, which suggested the result might be reliable. No significant publication bias was observed in funnel plot and Egger's test (Figure 4(d)). Chikata's study [17] was not included in the quantitative synthesis, but the results of this study supported that GA can significantly improve contact force (CF) parameters and reduce postoperative recurrence. Therefore, we thought there is a significant difference between GA/deep sedation and CS in the mid long-term procedural success rate of CA.

### 3.6. Evaluation of the Complication Risks of Different Anesthetic Strategies

**Figure 6:**
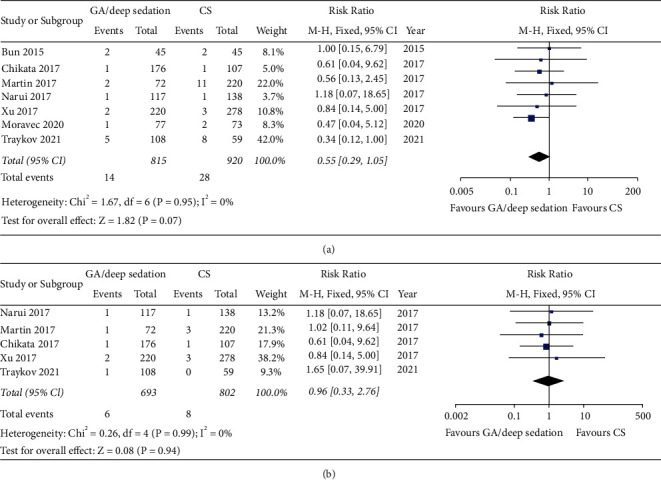
Forest plot comparing (a) total perioperative complications and (b) incidence of serious adverse events including atrioesophageal fistula, pericardial tamponade or pericardial effusion requiring puncture drainage between GA/deep sedation and CS in patients undergoing catheter ablation.

A total of seven studies reported complication rates post-AF ablation in patients using GA/deep sedation or CS. In total, the complication rate of the two groups was relatively low (1.7% in the GA/deep sedation group and 3.0% in the CS group). Although the former was slightly lower than the latter, the overall difference was not statistically significant (RR: 0.55, fixed-effect model, 95% CI, 0.29 to 1.05, *p*=0.07; Figure 6(a)). No heterogeneity between studies was observed (*I*^*2*^ = 0%, *p*=0.95 of *Q* test). However, the types of complications were different between the two groups. Most of the complications in the GA/deep sedation group were related to the endotracheal intubation or anesthesia process, such as postoperative cough, urinary tract infection, and intraoperative hypotension. Most of the complications in the CS group were related to agitation or with uncontrolled pain. Interestingly, there were no significant differences in serious adverse events including cardiac tamponade, atrial esophageal fistula or pericardial effusion requiring puncture drainage between the two groups (RR: 0.96, fixed-effect model, 95% CI, 0.33 to 2.76, *p*=0.94, *I*^*2*^ = 0%; Figure 6(b)). Result of the funnel plot and Egger' test showed no evidence of publication bias in terms of complications (Figures 4(e) and 4(f)).

### 3.7. Meta-Regression Analysis for Procedural Parameters and Clinical Outcomes

In this meta-analysis, procedural parameters had high heterogeneity, and a significant decrease in heterogeneity could not be observed through sequentially omitting one study at a time. Therefore, a meta-regression analysis was further conducted to find the influence of research characteristics on heterogeneity. Covariates included proportion of males, mean age, study type, publication year, research country, and anesthesia strategy. Regression analysis for recurrence rate also included follow-up duration. Specifically, countries are divided into Asia and Europe, study types were RCT and observational studies, anesthesia strategies are classified as GA vs CS and deep sedation vs CS. Results of the meta-regression analysis are shown in Table 3, and differences in these characteristics are not the factors affecting heterogeneity (*p* > 0.05).

## 4. Discussion

CA of AF can be performed either under different anesthesia methods, but it is still controversial [16, 24, 28]. This meta-analysis systematically compared the clinical benefits and safety of GA/deep sedation and CS by procedural time, recurrence rate, and adverse event risk. The main findings of it are as follows: (1) the use of GA/deep sedation may reduce the risk of mid long-term recurrence for patients who have undergone AF ablation compared with CS; (2) GA/deep sedation is the same as the CS approach in procedural and fluoroscopy time, but it was able to shorten the ablation time; and (3) it was equally safe between the two groups in terms of total complications and serious adverse event risks.

In terms of procedural time parameters, GA/deep sedation significantly reduced the ablation duration, possibly due to increased catheter stability during operation. It was considered that GA/deep sedation may reduce the time to adjust catheter position before each burning due to factors such as respiration and physical stability based on experience [17, 29]. Of note, there was no significant difference in total procedural time, which meant the additional time caused by the anesthesia factor may have accounted for the time in this discrepancy. According to previous studies reported, it may be due to the longer anesthesia induction and recovery time [24]. There was no statistical difference in the fluoroscopy time between the two groups after quantitative synthesis. However, the results among studies had great differences. With the development of three-dimensional electrophysiological mapping systems, fluoroscopy time is often related to catheter delivery and transseptal puncture, so patients' heart anatomy and operators' proficiency may cause the differences. Furthermore, only five studies reported mean fluoroscopy time, the small sample size may lead to sampling error. Overall, the anesthesia strategies may not affect those processes. Additionally, heterogeneity was high in all operative time parameters, which should be noted and discussed. For example, Wang's study [24] showed that the CS group had shorter procedural time, while Moravec's study [23] favored GA/deep sedation. According to check articles' descriptions, we did not find significant differences among studies. Sensitivity analysis was attempted but did not significantly reduce heterogeneity. Therefore, a meta-regression analysis was performed using all common covariates to explore possible associations between covariates and study outcomes, but the result did not reveal any covariates as a moderating factor for operative time parameters. Hence, we considered the high heterogeneity might be due to multiple reasons, including operator experiences, anesthesia depth during operation, and statistical heterogeneity caused by little of included studies.

Regarding the recurrence rates, this meta-analysis revealed that GA/deep sedation had a significant advantage in AF ablation. Studies of ablation parameters could explain this result. CA is usually a process that needs point-by-point burning for lesion areas to achieve electrical isolation at both ends of ablation line; gaps between burn points may cause CA failure, so it requires catheter to be placed accurately against the heart surface. However, this process is difficult due to the instability of the patient's breath, hemodynamics, and body. A previous study confirmed the benefit of ablation during apnea after comparing CF parameters during CA between apnea and ventilation [30]. Another study [16] showed that GA improved catheter stability and mapping system accuracy compared to CS, whereafter GA has been reported to improve CF parameters and force-time integral [31, 32]. These parameters were important for obtaining a permanent myocardial damage and considered to be strong predictors of conduction recovery and recurrences after ablation [33]. In our analysis, the results of one study using a remote magnetic navigation (RMN) system to ablation showed no significant differences in recurrence risks between different anesthesia strategies, which made our conclusions more convincing because RMN could increase catheter stability, thus partially offsetting the differences between GA/deep sedation and CS [14]. These findings implied that ablation under GA/deep sedation led to a more stable respiratory rhythm, increased ablation accuracy, reduced postoperative ablation target gaps formation and pulmonary vein reconnection, ultimately improving postoperative sinus rhythm maintenance rates.

In terms of procedural complications, GA/deep sedation could theoretically increase anesthesia-related complications, decrease adverse events due to operative pain, and might ultimately have no effect on total complication rates. This corresponded to the result of our analysis. Analysis limited to serious adverse events did not reveal any significant differences between GA/deep sedation and CS, which meant the two strategies were equally safe. Despite this, there are still some potential complications of GA/deep sedation that should not be ignored, such as nausea, vomiting, aspiration, throat pain, and urinary tract infection. For patients undergoing perioperative management is particularly important. The intensive chest pain caused by ablation during CS should also be concerned, which could cause uncontrolled movements and even lead to negative affect for procedures.

## 5. Limitation

Firstly, the number of studies included was not large enough, and most studies were not randomized controlled trials, so there were inevitably some variables between the two groups, such as techniques and population, that might potentially lead to bias of analysis. Secondly, high heterogeneity of included studies was found for the mean procedural time, ablation time, and fluoroscopy time, those results should be interpreted with caution. Finally, due to the limited available research results, some operation parameters such as mean anesthesia time, CF parameters, and ablation index cannot be adequately analyzed, although those parameters could more intuitively reflect the impact of different anesthesia strategies on the ablation process.

## 6. Conclusion

In the comparison of anesthesia strategies for AF ablation, there are no significant differences between GA/deep sedation and CS in terms of procedural time, fluoroscopy time, total complications, or incidence of serious adverse events. GA/deep sedation is associated with a shorter ablation time and a lower recurrence rate.

## Figures and Tables

**Table 1:** Quality assessment of observational studies.

Study	Publication year	NOS score
Traykov [25]	2021	7
Chikata [17]	2017	9
Wang [24]	2021	7
Xu [15]	2017	9
Bun [14]	2015	8
Martin [13]	2018	8
Narui [26]	2017	9
Hama [27]	2017	7

**Table 2:** Summary of included studies.

Study	Publication year	Country	Study type	Sample size	AF type	Male gender, *n* (%)	Mean age	Anesthesia strategy	Median followup duration (months)
Moravec [23]	2020	Czech republic	RCT	150	PAF	105 (70.0)	56.6	GA vs. CS	12
Stašková [22]	2017	Czech republic	RCT	50	PAF	33 (66.0)	59.8	GA vs. CS	12
Traykov [25]	2021	Bulgaria	Retrospective study	167	PAF, PsAF	116 (69.5)	57.5	GA vs. CS	20
Chikata [17]	2017	Japan	Retrospective study	176	PAF, PsAF	134 (76.1)	66.2	GA vs. CS	17.3 vs. 11.3
Wang [24]	2021	China	Retrospective study	351	PAF, PsAF	208 (59.3)	62.4	GA vs. CS	12
Xu [15]	2017	China	Retrospective study	498	PAF, PsAF	317 (63.7)	60.6	GA vs. CS	12
Bun [14]	2015	Monaco	Retrospective study	90	PAF, PsAF	63 (70.0)	60.5	GA vs. CS	12
Martin [13]	2018	UK	Prospective study	292	PsAF	238 (81.5)	59.2	GA vs. CS	12
Narui [26]	2017	Japan	Retrospective study	255	PAF, PsAF	229 (89.8)	56.5	Deep sedation vs. CS	18
Hama [27]	2017	Japan	Retrospective study	389	PAF	262 (67.4)	67	GA/deep sedation vs. CS	6

AF = atrial fibrillation; UK = United Kingdom; RCT = randomized controlled trial; PAF = paroxysmal atrial fibrillation; PsAF = persistent atrial fibrillation; RFA = radiofrequency ablation; GA = general anesthesia; CS = conscious sedation.

**Table 3:** Results of the meta-regression analysis for procedural parameters.

Slope coefficient	Standard error	Z value	*P* value	95% CI	
				Lower limit	Upper limit
*Procedural time*					
3.80313	27.57992	0.14	0.913	−346.633	354.2392
−20.06846	18.39054	−1.09	0.472	−253.7424	213.6055
−2.883939	3.674606	−0.78	0.576	−49.57424	43.80636
−436.7515	165.4476	−2.64	0.231	−2538.962	1665.459
7.288352	4.523885	1.61	0.354	−50.19305	64.76976
109.8325	62.75476	1.75	0.330	-687.5424	907.2074
*Ablation time*					
0.7524953	9.018126	0.08	0.939	−27.94721	29.4522
6.843837	8.885825	0.77	0.497	−21.43482	35.1225
−1.469066	1.819742	−0.81	0.479	−7.260297	4.322165
0.552666	55.07902	0.01	0.993	−174.7334	175.8387
−0.8592097	1.382002	−0.62	0.578	−5.257357	3.538938
−6.715246	10.64719	−0.63	0.573	-40.59935	27.16885
*Fluoroscopy time∗*					
−3.022216	3.156895	−0.96	0.514	−43.13437	37.08994
172.1302	199.2275	0.86	0.546	−2359.295	2703.556
−13.78631	24.25556	−0.57	0.671	−321.9825	294.4099
*Recurrence rate*					
−0.112459	0.1209298	−0.93	0.523	−1.649018	1.4241
0.2239085	0.4280696	0.52	0.693	−5.215231	5.663048
1.252249	1.027552	1.22	0.437	−11.80404	14.30854
0.0054298	0.0711709	0.08	0.952	−0.8988824	0.909742
−6.004763	4.139268	−1.45	0.384	−58.59914	46.58962
−0.2981956	0.2125513	−1.4	0.394	−2.998917	2.402525
0.808037	0.8573068	0.94	0.519	−10.08508	11.70115

^∗^Since only one study was different from the others, the study type, publication year, and anesthesia strategy had been tested by sensitivity analysis, so they were not included in this meta-regression.

## Data Availability

All the included studies data used to support the findings of this study are included within the article and have DOI numbers in the references.
